# Prenatal nicotine exposure and child behavioural problems

**DOI:** 10.1007/s00787-014-0615-y

**Published:** 2014-09-21

**Authors:** Carla M. T. Tiesler, Joachim Heinrich

**Affiliations:** 1 Helmholtz Zentrum München, German Research Centre for Environmental Health, Institute of Epidemiology I, Ingolstaedter Landstrasse 1, 85764 Neuherberg, Germany; 2 Institute for Medical Informatics, Biometrics and Epidemiology, Ludwig-Maximilians-University of Munich, Ingolstädter Landstraße 1, Neuherberg, Germany

**Keywords:** Tobacco smoke, Attention-deficit/hyperactivity disorder, Conduct problems, Depression, Anxiety, Brain development

## Abstract

In utero exposure to tobacco smoke has been related to numerous adverse health effects in new-borns, infants, children, adolescents and adults. The aim of this review was to summarise findings on prenatal nicotine exposure and its relationship with behavioural problems in the offspring. The majority of studies, and especially several recent epidemiological studies, observed a higher likelihood for attention-deficit/hyperactivity disorder (ADHD) or ADHD symptoms in exposed subjects. However, both human and animal studies have failed to provide clear evidence on causality. Existing literature on studies investigating the association between prenatal nicotine exposure and conduct or externalising problems in the offspring suggests a causal effect. The establishment of a final conclusion concerning the relationship between prenatal nicotine exposure and internalising problems in the offspring is complicated by insufficient data and mixed results in epidemiological studies. Prenatal nicotine exposure has been associated with altered brain structure and function in human offspring, and a proposed biological mechanism is related to nicotine’s adverse influence on neurotransmitter systems during brain development. In conclusion, establishing a statement on the causality of the relationship between prenatal nicotine exposure and behavioural problems in children remains a challenging task. Nevertheless, considering the results of an increasing number of studies which link prenatal exposure to nicotine to externalising problems applying different methodologies to account for confounding and in view of other adverse health effects known to be caused by this exposure, parents should consider smoking cessation.

## Introduction

Maternal smoking during pregnancy has been related to multiple adverse effects including pregnancy complications and risks of preterm delivery, lower birth weight, reduced lung function in infants and sudden infant death syndrome [1]. Another field of research links in utero exposure to tobacco smoke to behavioural problems such as attention-deficit/hyperactivity disorder (ADHD), conduct problems, depression and anxiety in the offspring.

The Global Burden of Disease study from 2010 estimated that mental and behavioural disorders cause around 185 million disability-adjusted life years (DALYs) [2]. Estimates for behavioural disorders in childhood were 5.8 million DALYs for conduct problems and half a million for ADHD [2]. However, even though a large number of studies support a relationship between intrauterine exposure to tobacco smoke and later behavioural problems and plausible biological mechanisms exist, some studies reported no association or only a weak one. Furthermore, many lifestyle, socioeconomic, cultural and genetic factors, in which smokers differ from non-smokers in non-random ways, complicate this relationship and make it difficult to come to a final conclusion on causality. Nicotine is just one component of tobacco smoke and smokeless tobacco products. However, there is increasing evidence for specific effects of prenatal nicotine exposure that leads to adverse health effects in new-borns, infants, children, adolescents and adults.

The objective of this review was to provide an overview on prenatal nicotine exposure and its relationship with offspring behaviour. As a broad range of symptoms and disorders is summarised under this term, we will summarise findings from previous reviews and complement them with relevant information from recent publications in the respective fields of research. First, main pathways of nicotine exposure during pregnancy are outlined. Then, the epidemiology of maternal smoking during pregnancy is covered including factors affecting the likelihood of smoking cessation. Subsequently, nicotine metabolism, methods of assessing prenatal exposure to nicotine and physical findings associated with this exposure are briefly described. The main section covers the specific topic of offspring behavioural problems following prenatal nicotine exposure. This section is subdivided into an overview of the literature covering (1) hyperactivity or inattention problems such as ADHD, (2) externalising problems such as conduct disorder and antisocial behaviour and (3) emotional or internalising problems such as depression and anxiety. Lastly, results from brain imaging studies and possible mechanisms of adverse effects of nicotine to the developing brain are discussed, followed by a final conclusion.

## Definition of prenatal exposure to nicotine and its assessment

Active maternal smoking is just one pathway of foetal exposure to nicotine. Others include an exposure of the pregnant woman to environmental tobacco smoke (ETS) due to smoking behaviours of other people, maternal use of smokeless tobacco products such as chewing tobacco and maternal use of non-tobacco products containing nicotine such as medication during a nicotine replacement therapy (NRT). ETS consists of mainstream smoke exhaled by the smoker and sidestream smoke which is released by the smouldering cigarette [3]. Tobacco smoke itself is a complex mixture of gases and particulate matter components [3]. One of these constituents is nicotine. Furthermore, prenatal exposure to nicotine is often difficult to quantify as nicotine concentration in different tobacco products varies and is also dependent on individual smoking characteristics such as puffing intensity [4].

To date, human studies, which analysed the association between prenatal nicotine exposure and behavioural problems, have defined this exposure either by active maternal smoking or by ETS exposure of the mother during pregnancy.

### Nicotine metabolism and its transfer from mother to the foetus

Nicotine is a chemical compound, an alkaloid, found in tobacco smoke, smokeless tobacco products and nicotine replacement products [5]. It can be absorbed through the mouth by chewing, the lungs as smoke or the skin via an NRT patch [5]. Once nicotine has entered the bloodstream, it is distributed throughout the body to various tissues such as the brain, lung and liver [5]. In the liver, it is metabolised and finally excreted in urine [5]. Nicotine’s major metabolite is cotinine whose main metabolic product in turn is *trans*-3′-hydroxycotinine [5]. By crossing the placental barrier, nicotine can be transferred from the maternal circulation to the foetus [6]. Cotinine measurements taken in the first trimester indicate an accumulation in foetal fluids: cotinine concentrations in amniotic fluid and foetal serum were higher than in maternal serum regardless of whether the mother is an active or passive smoker [7]. The main pathway of elimination of nicotine from the foetal circulation occurs by re-diffusion across the placenta into the maternal circulation [8].

### Assessment of prenatal exposure to nicotine

Prenatal exposure to nicotine can be assessed via questionnaires asking, for example, about maternal smoking or ETS exposure during pregnancy. This method is often chosen for epidemiological studies as past, long-term and current exposures can be inexpensively assessed in large samples [3, 9]. However, this method has several drawbacks. First, this method is prone to exposure misclassification such as recall bias, under-reporting or concealment of smoking, which might be due to awareness of numerous negative health effects for children exposed to tobacco smoke in utero [3, 9]. Second, a single question on whether the mother smoked during pregnancy does not adequately address the amount of exposure and smoking patterns: a “yes” answer might include total abstinence after becoming aware of the pregnancy. Therefore, a detailed set of questions on numbers of cigarettes smoked per day during pregnancy or during certain trimesters and on smoking cessation should preferably be used.

A more objective method of ascertaining prenatal exposure is the measurement of exposure-specific biomarkers in biological matrices of mother and/or child. Candidate biomarkers with high specificity for active or passive tobacco smoking or usage of medication containing nicotine are nicotine itself and its metabolites, such as cotinine or *trans*-3′-hydroxycotinine [10, 11]. While nicotine has a short half-life of 2 h, the half-life of cotinine is on average 16 h [10]. Thus, measurements of nicotine or cotinine in blood, urine and saliva reflect recent exposures within the last few hours or last few days, respectively, and are susceptible to variations in exposure. This limits the usage for the assessment of long-term exposures. In new-borns, however, a longer half-life of nicotine and similar half-life of cotinine compared to adults have been reported which have been suggested to be due to differences in the sensitivity of the clearance rates of these two chemicals to alterations of hepatic blood flow [5]. Maternal matrices such as blood, urine, saliva and hair can be used for detection of the above-mentioned biomarkers to assess ETS exposure or active smoking [10, 12]. Llaquet et al. [13] provide an overview on possible biological matrices in the child for detection of prenatal ETS exposure. Measurements of nicotine or its metabolites in cord serum and neonatal urine reflect the exposure to ETS shortly before delivery [13]. Other matrices such as hair, nails, amniotic fluid and meconium, in which nicotine or its metabolites accumulate or are incorporated during formation, have longer detection periods ranging from first to last trimester [13]. It is often not feasible to differentiate clearly between children of mothers exposed to ETS during pregnancy and those who were not. Two studies [14, 15] reported no detectable levels of nicotine or its metabolites in amniotic fluid or meconium of new-borns whose mother was exposed to ETS during pregnancy. In another study, the measurement of *trans*-3′-hydroxycotinine (adjusted for creatinine) in urine of new-borns showed slight but significant differences between children of mothers exposed to ETS and those unexposed [16]. For detection of foetal tobacco smoke exposure in late pregnancy, the measurement of cotinine in cord serum seems to be the most promising biomarker due to its ability to distinguish between active maternal smoking, maternal ETS exposure and no exposure [13].

Lastly, indoor nicotine concentrations can be measured in the homes of pregnant women using active or passive air samplers [17, 18]. This approach is mainly relevant for the assessment of ETS exposure.

## Epidemiology

Epidemiological studies using data from European birth cohorts reported maternal smoking prevalences between 14 and 38 % [19–24]. The European Perinatal Health Report from 2010 estimates the prevalence of maternal smoking during pregnancy or in the last trimester to be above 10 % in many countries, varying between under 5 % in Sweden and Lithuania to 19 % in Scotland [25]. Compared to the report from 2004, a slight decrease of about 1–3 % of mothers who smoked in the third trimester was observed [25]. Cnattingius [26] reviewed the epidemiology of smoking during pregnancy and also concluded that the prevalence had declined. While one-fourth and one-fifth of pregnant women in Sweden and the USA, respectively, reported smoking around 1990, these values declined to about one-eighth in 2000. Denmark showed slightly higher but similarly declining rates of approximately one-third at the beginning and one-fourth at the end of the nineties [26]. In 2004–2005, Bloch et al. [27] conducted a study on tobacco use and ETS exposure of pregnant women in nine developing countries. Percentages of women who reported active smoking ranged from 3 % (Pakistan) to 18 % (Uruguay). Estimates for ETS exposure at home ranged between 17 % (Democratic Republic of Congo) and 92 % (Pakistan) [27].

A wide range of smoking cessation rates during pregnancy was reported with 27–47 % in Europe, 23–43 % in the USA, 62–70 % in Japan and 4–47 % in other countries [28].

However, in spite of declining prevalences of smoking during pregnancy in most developed countries, and potentially increasing prevalences in some developing countries, exposure to nicotine during pregnancy is still a problem worldwide.

Another important aspect of the epidemiology of smoking during pregnancy is the constellation of factors associated with likelihood of quitting. Chances of quitting are lower in mothers with lower social status, smoking partner, higher degree of addiction, higher parity [28] and psychological correlates such as a history of conduct problems in childhood [29].

Finally, since most epidemiological studies used questionnaire-based exposure assessment scenarios, it has to be kept in mind that the reported findings cannot be interpreted as effects of pure nicotine exposure but rather as those of tobacco smoke, which, apart from nicotine, contains numerous other toxicants.

## Physical findings

Active maternal smoking is associated with a wide range of adverse health effects on the new-born. It is known to decrease birth weight, following a dose–response relationship [30, 31]. Other effects include increased risks for pregnancy complications and preterm delivery (<37 weeks of completed gestation), low birth weight (<2,500 g) and sudden infant death syndrome [1]. Maternal exposure to ETS during pregnancy is related to a similar range of outcomes. Salmasi et al. [32] conducted a meta-analysis on perinatal findings related to ETS exposure in pregnant women. Significant associations were found between ETS exposure and a lower birth weight, congenital anomalies and a longer birth length. ETS-exposed children further showed trends for smaller neonate head circumferences and risk for low birth weight [32]. Leonardi-Bee et al. [33] reported an increased risk for stillbirth of non-smoking women exposed to ETS while pregnant.

Nicotine exposure during pregnancy has been related to adverse effects on the lung and the respiratory system, including increased risks for asthma, wheeze and airway hyper-responsiveness in children [34–36]. Out of other components contained in tobacco smoke, nicotine has been suggested to be the main candidate concerning negative effects on pulmonary development [34]. Prenatal exposure to nicotine is related to structural as well as functional alterations in lung development, which are potentially related to an increased risk for obstructive lung disease and accelerated lung ageing in later years [34]. Finally, as prenatal nicotine exposure is a risk factor for low birth weight and as low birth weight is associated to adverse effects on lung development, nicotine might be indirectly related to adverse pulmonary development via low birth weight [37].

Bakker et al. [38] reviewed cardiovascular and metabolic influences of foetal smoke exposure. They reported that maternal smoking during pregnancy is associated with higher blood pressure in children. Furthermore, prenatal exposure to maternal smoking may directly or indirectly (via low birth weight) be related to obesity, adverse cardiovascular diseases and type 2 diabetes in later life [38]. While the association between maternal smoking during pregnancy and overweight or obesity in the children is suggested to be causal, no definitive conclusions for the outcomes of type 2 diabetes and the metabolic syndrome can be drawn from the small number of studies [39]. Moreover, there seems to be no association between smoke exposure during pregnancy and type 1 diabetes [39]. In short, findings for cardiovascular and metabolic effects of foetal smoke exposure are mixed and establishing statements about causality therefore stays difficult.

## Neuropsychological, behavioural and psychiatric findings

Prenatal nicotine exposure is also associated with increases in behavioural and cognitive problems.

Briefly, no definitive conclusion can be drawn regarding the association between prenatal exposure to nicotine or tobacco smoke and impairment of cognitive function in the children [31, 40, 41]. Differing results may be explained by incomplete control for confounding variables such as maternal age, education, intelligence quotient and socioeconomic status [31]. Clifford et al. [41] reviewed observational studies between 2000 and 2011 on the association between active maternal smoking during pregnancy and cognitive outcomes in children; they concluded that the most consistent results were observed for reduced academic achievement and impaired intellectual abilities. Animal studies on the effects of developmental nicotine on cognitive function in offspring show a similarly inconclusive picture with conflicting results [31].

Two very recent studies in children from the Avon Longitudinal Study of Parents and Children (ALSPAC), a prospective UK birth cohort, related prenatal nicotine exposure to impaired reading performance [42] and increased risk of language impairment and poor performance on language tasks [43].

The main objective of this review was to provide an overview on the topic of prenatal nicotine exposure and child behavioural problems subdivided into attention, externalising and internalising problems. Literature search was performed between November 2013 and January 2014. Initially, the PubMed database was searched by using the following terms: (“nicotine” OR “tobacco” OR “cigarette” OR “smoking”) AND (“prenatal” OR “pregnancy” OR “gestational” OR “trimester” OR “in utero”) AND (“neonate” OR “infant” OR “child” OR “children” OR “adolescent”) in combination with keywords for (1) ADHD and symptoms of hyperactivity or inattention (“attention-deficit/hyperactivity disorder” OR “ADHD” OR “hyperactivity” OR “inattention”), (2) conduct or externalising behaviours and antisocial behaviour (“conduct problems” OR “conduct disorder” OR “oppositional defiant disorder” OR “externalizing” OR “externalising” OR “aggression” OR “antisocial”) and (3) depression, anxiety and internalising disorders (“depression” OR “anxiety” OR “internalizing” OR “internalising” OR “emotional problems” OR “emotional disorders”). Further articles were identified via reference lists from earlier review articles.

Due to the large number of publications published to date in this field, this review will provide a comprehensive but not exhaustive overview on the current knowledge by summarising the results of previous review articles combined with findings from key publications and relevant recent publications in the respective fields of research. Details of the original publications mentioned in the text can be found in Table 1.

**Table 1:** Details on original publications stated in the main text analysing the relationship between prenatal nicotine exposure on offspring behavioural problems (subdivided into publications on attention, externalising or internalising problems shown in chronological order)

References	Study name (location of recruitment), Country	Number of participants	Age (years)^a^	Assessment of child’s exposure to smoking in utero; coding of exposure variable; % smoking [m]others or exposed [c]hildren, if different	Assessment of symptoms	Covariates	Results
**ADHD and symptoms of hyperactivity or inattention**							
Gatzke-Kopp [48]	Seattle, USA	133 Mothers and their 171 children	7–15	MSDP: reported; (a) no/yes/only ETS exposure; 16 % [m] smoked, 12 % [m] ETS (b) score based on packs/day summed across trimesters	CBCL and child symptom inventory	Household income, maternal and paternal antisocial symptoms, child BW, child GA at birth, maternal use of alcohol and other substances during pregnancy	Children exposed to MSDP or whose mother was exposed to second hand smoke during pregnancy had significantly higher ADHD symptom scores a higher MSDP score was associated with more ADHD symptoms
D’Onofrio [52]	Offspring of National Longitudinal Survey of Youth (NLSY79), USA	8,889	4–10	MSDP: reported; 0/0.5/1.5/2.5 packs per day; 36 %	Behaviour problem index (subset of items from CBCL): 3 items for ADHP	Maternal intellectual ability, maternal years of education, income, maternal delinquency, maternal age at first birth, child gender	MSDP was related to ADHP when exposed offspring was compared to unrelated offspring who was not exposed to MSDP MSDP was only related to small, non-significant effect on ADHP when siblings who were differentially exposed to MSDP were compared
Thapar [51]	Offspring conceived with Assisted Reproductive Technologies, UK and USA	815	4–11	MSDP: reported; no/yes; 6 %	DuPaul ADHD scale	Child age and gender, multiple birth status, mother’s ADHD symptoms, father’s ADHD symptoms, family income	In related mother–child pairs, MSDP was associated with ADHD symptoms in unrelated mother–child pairs, MSDP was not associated with ADHD symptoms
Nomura [50]	New York, USA	214	3–4	MSDP: reported; no/yes; 18 % FSDP: reported; no/yes; 33 %	ADHD-Rating Scale-IV, Kiddie-SADS-PL	Gender, age, race and BW of child, maternal alcohol use during pregnancy, family SES, mother’s and father’s ADHD symptoms, partner smoking during pregnancy	MSDP associated with increased risk of diagnoses of ADHD and of comorbid ADHD and ODD, whereas FSDP had no influence
Tiesler [22]	LISAplus, Germany	1,654	10	MSDP: reported; no/yes; 14 %	SDQ hyperactivity/inattention	Sex, study centre, parental education level, maternal age at birth, TV/PC usage, single parent status	MSDP alone or in combination with child’s post-natal exposure to smoking at home was associated with hyperactivity/inattention problems
Langley [20]	ALSPAC, UK	8,324	7.5	MSDP: reported; no/yes; 18 % FSDP: reported; no/yes; 32 % MPSP^b^: reported; no/yes; 6 %	Development and well-being assessment	Sex, multiple births, mother’s education, family social class, ethnicity, mother’s alcohol use during pregnancy	MSDP and FSDP, where mother is a non-smoker, during pregnancy were associated with more ADHD symptoms no association between MPSP and ADHD symptoms
Keyes [49]	Child Health and Development Study, USA	1,752	10	MSDP: reported; no/yes [0,1–9,10–19,20+], 33 % PSDP: reported; no/yes [0,1–9,10–19,20+]; NA	8 Items on hyperactivity from a 100-item battery of child characteristics	Partner smoking during pregnancy, father’s education, manual job, maternal current smoking, paternal current smoking, offspring race/ethnicity, child gender, maternal cognition, maternal age and child age (in subgroup: maternal alcohol and coffeine use during pregnancy)	MSDP and PSDP were associated with higher hyperactivity score, but only the association with MSDP withstood adjustment for covariates the MSDP dosage variable indicated a higher risk for a higher hyperactivity score for those who smoked 10–19 or 20+ cigarettes than for those who smoked 1–9 cigarettes compared to non-smokers children exposed to PSDP but not MSDP showed no significantly increased hyperactivity scores
Silva [47]	Population-based, record linkage case–control study, Australia	1,688 Cases, 3,849 controls	<25 (age was a matching variable)	MSDP: record-based; no/yes; 19 %	Diagnosis with ADHD and prescription of stimulant medication	Cases and controls matched by gender, year of birth and SES maternal age, marital status, first pregnancy, complications of pregnancy, induced onset of labour, augmentation of labour, complications of labour, type of delivery, GA, BW, appropriate for GA, small for GA and large GA, child Apgar at 5 min	MSDP was associated with increased risk for ADHD in boys and girls
**Conduct or externalising behaviours and antisocial behaviour**							
Gatzke-Kopp [48]	Seattle, USA	133 Mothers and their 171 children	7–15	MSDP: reported; (a) no/yes/only ETS exposure; 16 % [m] smoked, 12 % [m] ETS (b) score based on packs/day summed across trimesters	CBCL and child symptom inventory	Household income, maternal and paternal antisocial symptoms, child BW, child GA at birth, maternal use of alcohol and other substances during pregnancy	Children exposed to MSDP or whose mother was exposed to second hand smoke during pregnancy had significantly higher CD and aggression scores higher MSDP score was associated with more CD symptoms after adjustment for covariates
D’Onofrio [52]	Offspring of National Longitudinal Survey of Youth (NLSY79), USA	8,889	4–10	MSDP: reported; 0/0.5/1.5/2.5 packs per day; 36 % [c]	Behaviour problem index (subset of items from CBCL): 7 for CP, 3 for ODP	Maternal intellectual ability, maternal years of education, income, maternal delinquency, maternal age at first birth, child gender	MSDP was related to CP and to ODP when exposed offspring was compared to unrelated offspring who was not exposed to MSDP when siblings who differed in their exposure to MSDP were compared, the effect for both CP and ODP was substantially reduced and not significant
Stene-Larsen [56]	Norwegian Mother and Child Cohort Study, Norway	22,545	1.5	MSDP: reported; 0/1–9/10 + cigarettes per day; 13 %	8 items from CBCL/1.5–5y (oppositional behaviour [3], aggressive behaviour [2] and overly active behaviour [3] )	Mother’s level of education, single parent, maternal depressed mood, maternal alcohol use during pregnancy, maternal current smoking, child sex, BW <=2,500 g, GA <=37 weeks	Heavy MSDP (10+ cig./day) but not light MSDP (1–9 cig./day) was associated with an increased risk for externalising behaviour in the offspring
Brion [19]	Cohort 1: Pelotas, Brazil Cohort 2: ALSPAC, UK	Cohort 1: 509 Cohort 2: 6,735	4	Cohort 1: MSDP: reported; no/yes; 30 % FSDP: reported; no/yes; 50 % Cohort 2: MSDP: reported; no/yes; 16 % FSDP: reported; no/yes; 32 %	Cohort 1: CBCL (externalising scale) Cohort 2: SDQ (conduct problems scale)	Maternal and paternal education, social class, family income, parental psychopathology, partner’s smoking during pregnancy, BW, GA, breastfeeding	MSDP was associated with conduct/externalising problems FSDP was less strongly associated with conduct/externalising problems (significant only in Cohort 2 and lost significance after adjustment for parental psychopathology)
D’Onofrio [58]	Offspring of National Longitudinal Survey of Youth (NLSY79), USA	2,694 Mothers and their 6,066 children	14–17 (repeated assessments)	MSDP: reported; 0/0.5/1.5/2.5 packs per day; 27 % [c]	Offspring antisocial behaviour: self-reported delinquency scale reported criminal convictions	Gender, birth order, maternal teenage childbearing, maternal alcohol use during pregnancy, maternal adolescent antisocial behaviour, maternal intellectual abilities, maternal educational attainment, maternal income, maternal history of binge-drinking, maternal history of alcohol abuse/dependence, maternal adolescent substance use, family race/ethnicity	MSDP was associated to more antisocial behaviour and an increased risk for criminal convictions when exposed offspring was compared to unrelated offspring who was not exposed to MSDP MSDP was not associated with antisocial behaviour or risk for criminal convictions when differentially exposed siblings were compared
Gaysina [59]	Study 1: Christchurch Health and Development Study, New Zealand Study 2: Early Growth and Development study, USA Study 3: Cardiff IVF Study, UK	Study 1: 1,124 Study 2: 310 Study 3: 842	Study 1: 6 and 7 Study 2: 4.5 and 6 Study 3: 4–10	MSDP: reported by birth mothers; 0/1–9/10 + cigarettes per day, Study 1: 33 % [50 %] in genetically [un]related mothers Study 2: 41 % Study 3: 6 % [4 %] in genetically [un]related mothers	Parent- and/or teacher-reported child CP Study 1: Rutter and Conners behaviour scales Study 2: CBCL (externalising scale), Children’s Behaviour Questionnaire Short Form (impulsivity scale) Study 3: SDQ	Child gender, ethnicity, BW, breast feeding, maternal age at birth, maternal education, family SES, family breakdown, placement age, parenting practices	MSDP (by birth mothers) was associated with increased offspring conduct problems in both genetically related and genetically unrelated mother–child pairs
O’Brien [57]	East Boston Family Study, USA	176	Three measurements (exact age NA), participant age 11–18	MSDP: repeated cotinine-corrected reports; 48 %	ODD and CD scales from Diagnostic Interview for Children Antisocial Behaviour Checklist	Age, maternal and paternal antisocial behaviour, harsh parenting	Significant gene (*DAT1*) × MSDP interaction for boys on latent externalising spectrum (*DAT1* 10*r* variant related to higher risk in presence of MSDP)
**Depression, anxiety or internalising disorders**							
Höök [65]	Hässleholm and Western Blekinge, Sweden	1,428 (3 years), 677 (5.5 years)	3 and 5.5	MSDP: reported; 0/1–10/>10 cigarettes per day; 16 %	CBCL/2–3 (age 3) CBCL/4–18 (age 5.5)	None	MSDP was not associated with offspring internalising problems at 3 or at 5.5 years
Indredavik [62]	Subgroup of NICHD Study of Successive Small-for-Gestational Age Births, Norway	84	14	MSDP: reported; no/yes; 38 %	Youth self-report, CBCL and teacher report form, internalising and externalising scales	Gender, BW, SES, maternal age, single parent, mother’s present use of alcohol, mother’s mental health	MSDP was associated with higher scores on the internalising and externalising scales
Ashford [61]	Longitudinal general population study, Netherlands	396	5, 10–11 and 18	MSDP: reported; <10/10+ cigarettes per day; 7 % (10+)	CBCL/4–18 (ages 5,10–11) and CBCL/6–18 at age 18, internalising and externalising scales	Mother ill before pregnancy, mother ill during pregnancy, mother hospitalised during pregnancy, complications at birth, premature birth, low BW, child sex and SES, maternal mental health, child social and attention problems maternal mental health, child social and attention problems	MSDP was associated with increased internalising and externalising problems across all ages, even after control for co-occurrence of the two outcomes, across ages 5 to 18
Brion [19]	Cohort 1: Pelotas, Brazil Cohort 2: ALSPAC, UK	Cohort 1: 509 Cohort 2: 6,735	4	Cohort 1: MSDP: reported; no/yes; 30 % FSDP: reported; no/yes; 50 % Cohort 2: MSDP: reported; no/yes; 16 % FSDP: reported; no/yes; 32 %	Cohort 1: CBCL (internalising scale) Cohort 2: SDQ (emotional symptoms scale)	Socioeconomic position (maternal and paternal education, social class, family income), parental psychopathology, partner’s smoking during pregnancy, BW, GA, breastfeeding	Neither MSDP nor FSDP were associated with emotional/internalising problems
Ekblad [64]	Population-based longitudinal register data, Finland	175,869	Inpatient episodes (up to 18–20), outpatient visits (9–11 to 18–20)	MSDP: reported; 0/<10/>10 cigarettes per day; 15 %	Children’s psychiatric diagnoses related to outpatient visits and inpatient care (classified by ICD-9 and ICD-10)	Child sex, GA, BW, 5-min Apgar score, maternal age, parity and psychiatric diagnosis before birth of child	MSDP was associated with an increased risk for any psychiatric diagnosis dose–response relationships were observed for MSDP and the risks of mood disorders or the risks of behavioural and emotional disorders occurring in childhood and adolescence in total (also for subgroup of hyperkinetic disorders and subgroup of disorders of conduct and emotions regarded separately)
Menezes [63]	Pelotas, Brazil	4,106	18	MSDP: reported; no/yes; 33 % PSDP: reported; no/yes; 50 %	Subjective happiness scale, mini-international neuropsychiatric interview (for depression)	Sex, family income, planned pregnancy, partner support during pregnancy, maternal alcohol use during pregnancy, type of delivery, maternal anxiety and depression symptoms, partner’s smoking during pregnancy	MSDP was associated with decreased happiness and an increased risk for depression PSDP was associated with decreased happiness

*ADHP* Attention-deficit/hyperactivity problems, *BW* birth weight, *CBCL* Child Behaviour Checklist, *CD* conduct disorder, *CP* conduct problems, *FSDP/PSDP* father/partner smoking during pregnancy, *GA* gestational age, *ICD* International Classification of Diseases, *MSDP* maternal smoking during pregnancy, *ODD* oppositional defiant disorder, *ODP* oppositional defiant problems, *SES* socioeconomic status, *SDQ* Strengths and Difficulties Questionnaire

^a^At time of outcome measurement

^b^Neither parent smoked but third person smoked at home or exposure to ETS at work

### ADHD and symptoms of hyperactivity or inattention

An association between gestational exposure to nicotine or tobacco smoke and ADHD in children has been reported from many studies; results were summarised in several reviews (e.g. [31, 40, 44–46]). Linnet et al. [45] reviewed 24 studies published between 1975 and 2002 investigating the relationship between prenatal maternal smoking and ADHD or ADHD symptoms in the children. The authors concluded that most studies reported an increased risk for the development of such problems in children of smoking mothers, some even showing a dose–response effect in the association. However, as there were several serious shortcomings such as methodological issues related to retrospectively collected data, rough estimation of exposure by a dichotomous smoke exposure variable and statistical issues related to power, no final statement on causality was possible [45].

Latimer et al. [44] reviewed prenatal or early post-natal environmental risk factors associated with disruptive behaviour disorders. Eleven studies investigated the role of maternal smoking during pregnancy: eight of them, including population-based and case–control studies of good quality, supported the presence of a link to an increased risk for ADHD in the offspring.

Furthermore, results from about 1,600 children of the German birth cohort study LISAplus also support an association between maternal smoking during pregnancy and hyperactivity or inattention problems in 10-year-olds [22].

In a population-based record linkage case–control study of young non-Aboriginal Australians (about 1,700 cases and 3,850 controls), Silva et al. [47] recently observed that maternal smoking is a risk factor for clinically defined ADHD with additional prescription of stimulant medication. The association remained significant for both sexes even after adjustment for several characteristics related to pregnancy and birth (boys: odds ratio (OR) = 1.86, 95 % confidence interval (CI): 1.53–2.27; girls: OR = 1.67, 95 %CI: 1.07–2.61).

Thus, both the consistency of results across many studies and different study designs and the presence of dose–response relationships between exposure and outcome in some studies support the hypothesis of a causal association. A further aspect is related to the ETS exposure of women who are non-smokers during pregnancy. This exposure can be due to ETS exposure at home by the partner or other household members or it can be an exposure at the workplace. Several studies have compared the effects of active maternal smoking during pregnancy with those resulting from ETS exposure (e.g. [20, 48–50]).

Gatzke-Kopp et al. [48] observed a higher risk for ADHD symptoms not only in children exposed to maternal smoking during pregnancy but also in those whose mother did not smoke but was exposed to ETS during gestation.

A very recent study by Keyes et al. [49] compared the influence of maternal and/or paternal smoking on offspring hyperactivity at the age of 10 years. In unadjusted analyses, maternal as well as paternal smoking during pregnancy was related to increased offspring hyperactivity, respectively. After adjustment for partner’s smoking behaviour and accounting for several covariates, the association between maternal smoking and hyperactivity in the children remained stable, but the association with paternal smoking was attenuated to non-significance. Furthermore, no increased risk for hyperactivity could be observed in children whose father smoked during pregnancy and whose mother did not smoke. Nomura et al. [50] conducted a similar study in about 200 preschool children (3–4 years old). They observed an increased risk of ADHD symptoms only for children exposed to maternal smoking but not for those exposed to paternal smoking, even after adjustment for a series of confounders including ADHD symptoms of the parents, thereby decreasing the chance for confounding by genetic factors.

Langley et al. [20] used data from over 8,000 children of the ALSPAC prospective birth cohort study. They compared the risks of ADHD symptoms in children aged 7.5 years whose mother smoked during pregnancy, with those whose mother did not smoke but was exposed to the smoking behaviour of the father. Furthermore, they assessed the effect of passive smoking in families in which neither parent smoked but where the mother reported ETS exposure at work or living with household members who smoked. Maternal smoking and paternal smoking (even in the absence of maternal smoking) were both observed to be associated with increased ADHD symptoms in the offspring, while passive smoking was not. The authors concluded that the associations between maternal smoking and ADHD in the children may be confounded by genetic factors or factors on the household level and are to a lesser extent attributable to causal effects of an exposure in utero [20].

Further studies that were able to control for genetic factors suggest that the association between maternal smoking during pregnancy and ADHD might not be causal (e.g. [51, 52]).

Thapar et al. [51] tested the association with maternal smoking in children conceived with assisted reproductive technologies, comparing the ADHD risk of children genetically related and unrelated to the gestational carrier. In genetically related mother–child pairs, maternal smoking during pregnancy was related to an increased risk for ADHD symptoms in the offspring, while no association was observed for genetically unrelated pairs. This observation suggests that the effect might be rather attributed to inherited characteristics than to the exposure to prenatal smoking [51].

D’Onofrio et al. [52] compared the ADHD traits of siblings with and without prenatal maternal smoke exposure in order to account for familial and genetic effects. When children whose mother smoked during pregnancy were compared to unrelated children without prenatal smoke exposure, they showed a significantly increased risk for ADHD symptoms. However, in siblings who differed in their exposure to maternal smoking during pregnancy, the association between smoking and subsequent ADHD symptoms was small and not significant.

Abbott et al. [31] provided an overview on findings from animal studies exploring whether prenatal exposure to tobacco smoke is associated with ADHD-like symptoms. Most studies used nicotine instead of tobacco smoke and hyperactivity measured by increased locomotor activity was usually chosen as indicator for ADHD symptoms. Studies in mice mostly reported increased locomotor activity after prenatal nicotine exposure, but studies in rats were less consistent [31]. Furthermore, the authors stated that increased activity might not be representative of ADHD-like behaviour in rodents, as other symptoms such as inattention are not considered [31]. Moreover, several methodological issues complicate the transfer of results from rodent studies to humans. Dwyer et al. [53] mention three caveats: first, human foetuses are born at a more mature stage of brain development than are rodents. The first two trimesters of human development correspond approximately to the full gestational development of rodents, and the early post-natal period of rodents is used as model for the third trimester development of human foetuses. Second, the effects of a continuous exposure to nicotine as effected in rodent models might be different from an intermittent exposure related to variations in nicotine levels such as it is the case for human smoking. Third, animal models with a nicotine exposure do not reflect the exposure to tobacco smoke in humans as tobacco smoke contains numerous other chemicals besides nicotine [53].

In summary, the majority of studies, and especially several recent epidemiological studies, observed a higher likelihood for ADHD or ADHD symptoms in subjects prenatally exposed to nicotine. However, both human and animal studies have failed to provide clear evidence on causality.

### Conduct or externalising behaviours and antisocial behaviour

Several review articles (e.g. [54, 55]) summarised the association between prenatal exposure to nicotine and conduct or externalising problems. The authors concluded that the existing literature strongly indicates an increased risk, but no causal association could be established due to methodological limitations.

Further support for an increased risk comes from the study of Gatzke-Kopp et al. [48] who observed that not only active maternal smoking, but also non-smoking mother’s ETS exposure, during pregnancy is related to higher symptom scores for conduct disorder in the offspring.

Two other studies investigated the association between maternal smoking during pregnancy and increased risk for externalising problems in relatively young children at an age of 18 months [56] and 4 years [19]. Using data from the population-based Norwegian Mother and Child Cohort Study with a large study sample (*N* > 22,500), Stene-Larsen et al. [56] reported a significantly increased risk for externalising problems for 18-month-old children whose mother smoked more than 10 cigarettes per day (OR = 1.32, 95 % CI: 1.03–1.70) but not for those who smoked less. The authors additionally reported no sex difference in the association [56]. Brion et al. [19] analysed the association of maternal smoking during pregnancy with conduct or externalising problems in 4-year-olds from two birth cohorts, one from a middle-income country (Brazilian Pelotas study) and one from a high-income country (British ALSPAC study). A significant effect was (1) present in both studies, (2) persisted even after adjustment for confounders such as socioeconomic status and parental psychopathology and (3) was also robust to adjustment for paternal smoking during pregnancy.

Recently, O’Brien et al. [57] reported a gene x environment interaction for a dopamine transporter gene (*DAT1*) variant that modifies the risk for externalising problems in male but not in female adolescents after prenatal exposure to maternal smoking which was assessed via repeated cotinine-corrected reports.

However, studies using specific designs to control for genetic confounding came to inconsistent results (e.g. [52, 58, 59]).

D’Onofrio et al. [58] recently assessed the relationship between maternal smoking during pregnancy and antisocial behaviour in adolescents aged 14–17 years. In unrelated individuals, the results show a significantly increased risk for antisocial behaviour symptoms and for a criminal conviction. However, the associations became smaller and lost statistical significance when comparing siblings who differed in their exposure to prenatal maternal smoking. This supports the influence of familial factors on the association between prenatal exposure to smoking and later development of antisocial behaviour. A similar result was observed in an earlier study from D’Onofrio et al. [52] who also did not observe any elevated risk for conduct problems or oppositional defiant problems in children exposed to prenatal maternal smoking compared to unexposed siblings.

However, a very new study by Gaysina et al. [59] shows a different picture. Using data from three studies, it supports a direct causal effect of prenatal maternal smoking on later conduct problems in the offspring. Three different genetic constellations for mother–child pairs were present: either genetically related (1) or genetically unrelated with an adoption of the child at birth (2) or at conception (3). Thus, the authors were able to assess the association in mother–child pairs who differed not only with respect to tobacco smoke exposure during pregnancy but also with respect to their genetic relationship. Children who were exposed to maternal smoking in utero were observed to have a higher risk of conduct problems, regardless of whether the mother was genetically related or unrelated to the child. This supports an adverse effect of the exposure and not of genetic factors. This association was also observed after adjustment for several potential confounders among which were maternal education, parenting practices and socioeconomic characteristics of the family. Furthermore, results of a meta-analysis across pairs in the three studies supported this finding [59].

A possible explanation for differences between the findings from the study of Gaysina et al. [59] and those from D’Onofrio et al. [52, 58] is proposed by Gaysina et al.: the inability of the latter two studies to account for the influence of passive gene–environment correlations. Contrary to a gene–environment interaction which refers to a different susceptibility to an environmental factor due to a certain genotype, gene–environment correlations are described by a probability of exposure to an environmental factor that differs with the genotype [60]. A passive gene–environment correlation refers to the situation that children with a certain genotype (that is inherited from the parents) are more likely to experience a certain environmental exposure occurring during childhood [60]. Gaysina et al. [59] mentioned that they were able to control for post-natal passive genotype–environment correlations by testing the association in a subgroup of children adopted at birth who share the post-natal environment, but no prenatal environmental nor genetic factors with the mother. The association between prenatal smoke exposure and children’s conduct problems was also present in this group.

In conclusion, existing literature suggests a causal effect of prenatal exposure to nicotine and conduct or externalising problems in the offspring.

### Depression, anxiety or internalising disorders

Results are generally mixed on the association between maternal smoking during pregnancy and internalising symptoms, such as depression or anxiety, in children. Findings are therefore less consistent than the findings for externalising symptoms such as conduct problems or ADHD [61]. While some studies are supportive of a relationship (e.g. [61–64]), others are not (e.g. [19, 65]).

One longitudinal study by Ashford et al. [61] investigated the association between maternal smoking during pregnancy and symptoms of internalising behaviour in nearly 400 children, assessed at ages 5, 10–11 and 18 years. The authors observed significant relationships with both externalising and internalising behaviours that were also robust to adjustment for potential confounders and also for co-occurring internalising and externalising behaviours, respectively. The authors state that their study has the advantage of controlling for comorbid externalising problems, as the association between maternal smoking during pregnancy and externalising problems is well established and internalising and externalising problems are often comorbid.

A similar result was observed from a small study of 84 children conducted by Indredavik et al. [62]. Maternally reported internalising scores at the age of 14 years were significantly higher for children whose mother smoked during pregnancy, and this association remained also after adjustment for confounders including socioeconomic status and maternal mental health.

Menezes et al. [63] recently reported results from 18-year-olds from the Pelotas cohort, showing a higher risk for lower levels of happiness and increased rates of depression among those prenatally exposed to maternal smoking (<20 cigarettes/day: OR = 1.38, 95 % CI: 1.03–1.84; ≥20 cigarettes/day: OR = 2.11, 95 % CI: 1.31–3.40). Smoking by the mother’s partner during pregnancy was associated with decreased adolescent happiness after adjustment for confounders, but did not show an association with offspring depression.

Ekblad et al. [64] studied the relationship between maternal smoking during pregnancy and psychiatric morbidity in young Finnish adults in a large (*N* > 175,000) population-based sample using registry-based data. The authors observed an increased risk for any psychiatric diagnosis. Dose–response relationships were observed for the risks of mood disorders, behavioural and emotional disorders occurring in childhood and adolescence, as well as disorders of conduct and emotion. However, while the study was able to adjust for a potential influence of maternal psychiatric morbidity on the relationship, information on other important factors such as socioeconomic factors, maternal alcohol consumption during pregnancy and post-natal exposure to tobacco smoke was not available. Therefore, the reported association should be interpreted with caution.

Other studies, however, do not observe a relationship between prenatal maternal smoking and internalising problems. Höök et al. [65] observed no such association in preschool children at 3 or at 5.5 years of age. A similar null finding was observed by Brion et al. [19] who studied the association in the British ALSPAC and the Brazilian Pelotas study. Another finding of this study was that paternal smoking during pregnancy was also unrelated to offspring internalising problems [19].

A small number of studies in rats on internalising behaviours after prenatal nicotine exposure reported consistent results of an increased anxiety-like behaviour in adolescent and also in adult rats [31].

In summary, the establishment of a final conclusion concerning the relationship between prenatal nicotine exposure and internalising problems in the offspring is complicated by insufficient data and mixed results in epidemiological studies.

## Developmental aspects: neonate, infant, child, adolescent and adult

Taking together the results from above, maternal active or passive smoking during pregnancy correlates with behavioural problems in the offspring across the lifespan. However, even if no study had had the possibility to investigate the longitudinal association from birth to adulthood, the results from offspring at different ages seem to support a long-lasting relationship.

## Central nervous system (CNS) findings

Bublitz et al. [66] reviewed the results from the small number of studies investigating the association between maternal smoking during pregnancy and brain structure and function in human offspring, and concluded that this exposure has adverse effects. Structural changes in the foetal or early post-natal period included smaller volumes of cerebellum and lateral ventricular system and a smaller frontal lobe [66]. Structural changes in children and adolescents exposed to gestational tobacco smoke included reduced grey matter volume in the cerebral cortex, smaller volume of the corpus callosum and thinning in the frontal, temporal and parietal regions [66]. Reduced volume of the corpus callosum and cerebellum was also observed in children with ADHD [67], thereby providing a potential link between in utero exposure to tobacco smoke and ADHD. Furthermore, comparative functional magnetic resonance imaging studies showed inferior frontal cortex underactivation in children with ADHD which is suggested to be disorder-specific for ADHD [68]. Imaging studies of conduct disorders showed dysfunctions of the paralimbic system which were disorder-specific when compared to children with ADHD [68].

Bublitz et al. [66] reported an increased rate of auditory brainstem responses in infants whose mother smoked during pregnancy, providing a link to cognitive deficits or language and learning impairments.

## Mechanisms underlying teratogenesis and/or effects on the CNS

Nicotine is one of thousands of components of tobacco smoke, but it is this chemical that most probably has adverse effects on brain development [53]. The effects of nicotine are thought to occur via its action on nicotinic acetylcholine receptors (nAChRs). These receptors are ligand-gated ion channels expressed in the CNS, in the peripheral nervous system and also in non-neuronal cells that operate through binding and release of a signalling molecule which, in the case of nAChRs, is the endogenous neurotransmitter acetylcholine (ACh) [69]. Nicotine is an exogenous agonist of ACh, able to bind to and to desensitise these nAChRs and thereby mimics the action of ACh [53]. It is suggested that ACh through its action on nAChRs plays an important role in brain maturation in foetuses and infants up to adolescence [53]. These processes modulated by ACh can thus be perturbed by nicotine. Expression of nAChRs subunit mRNA is reported in the first trimester of human foetuses [70], and its expression varies among brain regions and time but seems to be comparable between humans and rodents [53]. Therefore, nicotine exposure at different periods of maturation could elicit different developmental deficits [53]. There is further evidence for the involvement of nAChRs in the control of CNS maturation by modulating, for example, gene expression, cell proliferation, differentiation and apoptosis [70]. Results from animal studies also suggest that gestational exposure to nicotine is related to cell death in neurons [70]. NAChRs are also thought to be involved in the development of the catecholamine neurotransmitter systems via regulation of neurotransmitter release [70]. This provides a link to behavioural problems in children as these may be a result from catecholaminergic dysfunction that could potentially be caused by nicotine that perturbed a proper development [53]. Arnsten and Rubia [68] reviewed the role of neurobiological circuits involved in the regulation of behaviour and cognitive function and their relationship to neurodevelopmental disorders in children. The prefrontal cortex (PFC) plays here an important role which is dependent on optimal levels of neurotransmitters such as the catecholamines dopamine and norepinephrine, serotonin and ACh [68]. A reduced function of the prefrontal cortex is related to symptoms of ADHD [71]. Treatment with methylphenidate, a stimulant medication for ADHD in children, increases the levels of dopamine and norepinephrine in the PFC by blocking transporters responsible for the clearance of these neurotransmitters and thereby improves the PFC’s function to control attention and working memory [68].

Moylan et al. [72] reviewed possible biological mechanisms that might be involved in the relationship between in utero tobacco smoke exposure and anxiety symptoms or disorder. Among these is a role of neurotransmitter systems, such as serotonin, noradrenaline and dopamine. As mentioned above, these systems might be affected by prenatal exposure to nicotine. Furthermore, dysfunction of norepinephrine and serotonin neurotransmitter systems has been related to depression and anxiety [73, 74].

Another approach for understanding the influence of maternal smoking on child behaviour problems is the investigation of epigenetic mechanisms [75, 76]. Prenatal smoking has been linked to alterations in placental DNA methylation and gene expression [77], decreased global DNA methylation in cord blood relative to cord serum cotinine levels [78] and to increased DNA methylation in the brain-derived neurotrophic factor-6 exon in adolescence [79]. However, the implications of these findings for behavioural outcomes in the offspring are not yet clear [75].

## Conclusion

Many studies report relationships between maternal smoking during pregnancy and behavioural problems or impaired cognitive function in the offspring, and plausible biological mechanisms exist via which nicotine could affect the development of the foetus in utero. The link of prenatal smoke exposure with externalising problems or ADHD seems to be more consistent than that with internalising problems. However, establishing a statement on the causality of the relationship between prenatal nicotine exposure and behavioural problems in children remains challenging.

But, do we really need more research on the direct causality between prenatal exposure to nicotine and behavioural problems to recommend abstinence from smoking during pregnancy and the avoidance of a smoke exposure of the expectant mother? We think that further studies are likely unnecessary! This final statement is not only justified by an increasing number of studies which link prenatal exposure to nicotine to externalising problems applying different methodologies to account for confounding but also by other adverse health effects known to be caused by prenatal nicotine exposure. Therefore, parents should consider smoking cessation to prevent adverse health effects for their child.

## Abbreviations

ACh: Acetylcholine
ADHD: Attention-deficit/hyperactivity disorder
CI: Confidence interval
CNS: Central nervous system
DALY: Disability-adjusted life year
ETS: Environmental tobacco smoke
nAChR: Nicotinic acetylcholine receptor
NRT: Nicotine replacement therapy
OR: Odds ratio
PFC: Prefrontal cortex
